# Chloroplasts Use Calcium Signals to Call for Help under Heat Stress

**DOI:** 10.1093/pcp/pcz039

**Published:** 2019-02-22

**Authors:** Markus Teige

**Affiliations:** Molecular Systems Biology, Faculty of Life Sciences, University of Vienna, Vienna A-1090, Austria

Although chloroplasts perform the essential steps of carbon assimilation and initiate many biosynthetic pathways, they are increasingly recognized as critical sensors of environmental response in plants (Stael et�al. 2015, Chan et�al. 2016, Kmiecik et�al. 2016). It is known for quite some time that the photosynthetic machinery in chloroplasts is susceptible to different stresses, with photosystem II (PSII) in particular being very sensitive to thermal damage (Berry and Bjorkman 1980). Plants employ a number of different mechanisms to enhance photoprotection under diverse stress conditions (Pinnola and Bassi 2018). Many of these mechanisms are specifically tailored to avoid damage to PSII. Recently, the small chloroplast heat-shock protein 21, a major protection factor for PSII, was shown to be activated by a retrograde chloroplast-to-nucleus signaling pathway (Chen et�al. 2017). While in the green algae *Chlamydomonas reinhardtii*, calcium has been implicated in the regulation of cyclic electron flow—another of those photoprotective mechanisms—via a small calcium sensor protein named CAS (Terashima et�al. 2012), the role of calcium in chloroplast stress responses of higher plants is still unclear.

In this issue, Lenzoni and Knight (2019) show that increases in the absolute temperature elevate the free calcium concentration in chloroplasts (Lenzoni and Knight 2019). This response is specific to chloroplasts as a similar response is not detectable in the cytosol after a sudden shift from 20�C to 40�C (Fig.�1). Furthermore, the response was demonstrated to be dynamic, dose-dependent and dependent upon absolute temperature, not the rate of heating. Interestingly, a 30�C heat stimulus did not trigger a stromal calcium increase, indicating that this temperature presents a threshold for the chloroplast calcium heat response. When plants were exposed to consecutive heat stimulation of the same magnitude, the calcium signal showed attenuation, similar to the previously described cytosolic calcium signal upon cold treatments. However, in contrast to the cytosolic cold sensing, the chloroplast high temperature sensing seemed to be mostly dependent on the absolute temperature, rather than rate. Notably in this study, differences between different species could be observed. For example, tobacco required a temperature elevation to 45�C to trigger a calcium signal, which is comparable to that of Arabidopsis at 40�C. Mechanistically, the calcium signals were found to be partially dependent on the calcium-sensing CAS protein, which has been shown previously to regulate other chloroplast calcium signaling responses. Similar chloroplast calcium increases have been reported before in response to cold, salt and hyperosmotic stresses as well as pathogen elicitor molecules (Manzoor et�al. 2012, Nomura et�al. 2012). Thus, calcium is emerging to be an important stabilizer of the oxygen-evolving complex at PSII in addition to its involvement in regulatory and signaling events in chloroplasts; however, different mechanisms are clearly involved in the generation and regulation of calcium signals depending on the stimulus.

**Fig. 1:**
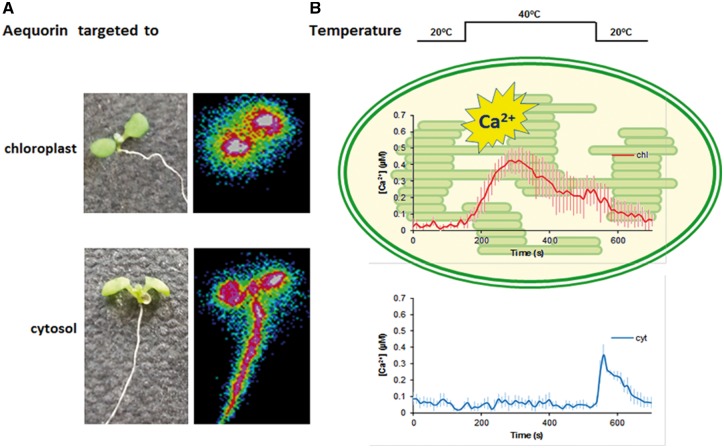
Detection of calcium signals in chloroplasts and in the cytosol upon temperature changes. (A) Representative image of seedlings (brightfield, left) expressing aequorin either in the chloroplast (upper panel), or in the cytosol (lower panel), and their total aequorin signal output (right panels) for the time measured (including discharge), showing that the signal only appears in plant shoots of the chloroplast-targeted line, and in the entire seedling for lines harboring the cytosolic aequorin. The colors represent the aequorin signal intensity (calcium signal) in pseudocolors using a scale ranging from cold (low signal) to warm colors (strong signal). (B) Quantification of the calcium signal in response to temperature shifts in the chloroplast and the cytosol corresponding to seedlings shown in (A).

## Disclosures

The authors have no conflicts of interest to declare.
